# libcov: A C++ bioinformatic library to manipulate protein structures, sequence alignments and phylogeny

**DOI:** 10.1186/1471-2105-6-138

**Published:** 2005-06-06

**Authors:** Davin Butt, Andrew J Roger, Christian Blouin

**Affiliations:** 1 Faculty of Computer Science, Dalhousie University, 6050 University Ave. Halifax, NS, B3H 1W5, Canada; 2 Dept. of Biochemistry and Molecular Biology, Dalhousie University, Tupper Medical Building, Halifax, NS, B3H 1X5, Canada; 3 Canadian Institute for Advanced Research (CIAR)

## Abstract

**Background:**

An increasing number of bioinformatics methods are considering the phylogenetic relationships between biological sequences. Implementing new methodologies using the maximum likelihood phylogenetic framework can be a time consuming task.

**Results:**

The bioinformatics library libcov is a collection of C++ classes that provides a high and low-level interface to maximum likelihood phylogenetics, sequence analysis and a data structure for structural biological methods. libcov can be used to compute likelihoods, search tree topologies, estimate site rates, cluster sequences, manipulate tree structures and compare phylogenies for a broad selection of applications.

**Conclusion:**

Using this library, it is possible to rapidly prototype applications that use the sophistication of phylogenetic likelihoods without getting involved in a major software engineering project. libcov is thus a potentially valuable building block to develop in-house methodologies in the field of protein phylogenetics.

## Background

With the development of genomics, research in biology and systems biology is becoming increasingly data-driven. The feedback between available data and hypotheses has accelerated the pace at which innovative ideas are generated. Life scientists are in a position to design novel methodologies but do not necessarily have the in-house skills to produce software implementations. Simple methods, made of complex building blocks such as maximum likelihood calculations, require major software development projects before they can be prototyped. The use of libraries can help to rapidly prototype software implementations.

We present libcov, an object-oriented library to perform phylogenetic inference and the manipulation of protein sequences and structures. The library is written in C++, is compliant with the GNU standards and packaged as a dynamic library that can be installed on most Unix distributions (including MacOS X).

There are other bioinformatic libraries available, many of which overlap with libcov in their functionalities. The PAL library, for example, [1] is a Java implementation which offers a versatile object set for nucleotide and protein phylogeny. More generally, interested readers can visit the Open Bioinformatics Foundation [2] that links to a series of libraries written in various popular scripting languages such as Perl and Python. Further, there are other libraries available in C++ such as the Bioinformatics Template library BTL [3], and the compBioTool++[4], both of which focus on sequence manipulation.

The scope of libcov is to offer a series of high-level functions that can be invoked in one line of code, and which does not force an implementation to adopt specialized custom types. As for any open source project, it is possible to use or extend the low-level Application Programming Interface (API) to add functionalities or entirely new modules.

## Implementation

Libcov offers a high-level programming interface, using an Object-Oriented (OO) approach with classes to represent distinct identities. For example, the class covTree represents a phylogenetic tree, covAlignment is used to store alignments, and class PDBentity handles 3D protein structures from the PDB format. The PDBentity class is a hierarchical structure of peptide chains, residues and atoms. Other classes handle elements such as geometric transformations and substitution matrices.

**Figure 1:**
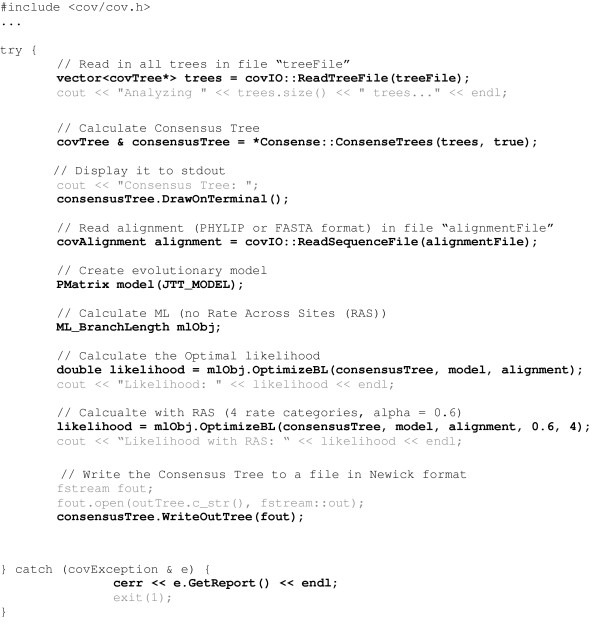
**The likelihood of a consensus tree. **In this example, a file containing trees in NEWICK format is parsed and a consensus tree is resolved using the greedy majority-rule consensus algorithm [9, 21]. Finally, the likelihood of the resulting tree is calculated. Bolded lines are libcov API calls.

Libcov is designed as a protein phylogeny library. The data structures and methods that its public interface offers can be integrated within application prototypes with a minimal impact on software design. Most of the return types are Standard Template Library (STL) containers, which can be seamlessly integrated into ongoing software projects. Specialized classes can be derived by consulting the online API documentation. Examples of integration of libcov within C++ source codes are presented in Figure 1.

A summary of the functions offered by libcov is presented in TABLE 1. A more complete list of methods is available at the project's website.

Currently, we have implemented three major applications using libcov. covTREE is our protein sequence simulator that has the ability to simulate complex patterns of protein evolution and phylogenetic artifacts[5]. It uses the Monte Carlo-based simulation functions that libcov provides. covSEARCH is a tree searching program using the maximum likelihood and tree re-arrangement algorithms in libcov. covARES maps sequence and phylogenetic information on to protein models [6]. These applications are also available to the research community under a GNU GPL license.

## Conclusion

The libcov library is actively under development, and we will be frequently releasing updated versions. As libcov is the engine powering the phylogenetic application covSEARCH, future work will involve new algorithms of tree searching, confidence interval determination and the integration of structure-based models of substitution.

External contributions are welcomed as the functionality of the library will evolve to match the research interests of the developers of phylogenetics applications.

## Availability and Requirements

Project's name: libcov

Project's website: 

Operating System: GNU C++ library. Tested on Linux, MacOSX and other Unix-based operating systems.

License: GPL

Non-academic licensing: None.

## Authors' contributions

**C. Blouin **– Scientific Functionalities, High-level design, Redaction of manuscript.

**D. Butt **– Software design, implementation and testing.

**A.J. Roger **– Scientific functionalities, redaction of manuscript.

## Figures and Tables

**Table 1:** High Level functionalities

**Category**	**Method**	**Reference**
I/O	Tree (NEWICK) Sequences (FASTA, PHYLIP) Protein Structure (PDB)	
Tree manipulation	Random/exhaustive: Subtree Pruning Regrafting (SPR) Tree Bissection Reconnection (TBR) Nearest Neighbor interchange (NNI) Branch Swapping Stepwise addition	[7]
Phylogeny	Neighbor Joining	[8]
	Greedy Majority-rule consensus	[9]
	Maximum Likelihood Rates across site modeling Estimation of shape parameter α	[10]
ML confidence intervals	KH	[11]
	SH	[12]
	RELL	[13]
	Expected Likelihood Weights	[14]
ML performance	P-matrix caching Chebyshev Polynomial approximation	[15]
Substitution matrices	JTT	[16]
	PAM	[17]
	WAG	[18]
Simulation	Protein Sequence Simulation (Rates across sites, rate shifts, site specific frequencies, multiple datasets, likelihood computation)	[5, 19]
	Random Number generation	[20] Acknowl. Z. Yang for implementation in PAML
Structural Biology	Manipulation / mapping Neighboring site anisotropy (NSA) Geometric transformations Distance/Contact Matrices	[6]
